# 

**Published:** 1889-08-03

**Authors:** 


SATURDAY, AUGUST 3rd, 1889.

**Lord Rosebery's Work** - - - 271
**Words of Consolation**.—The Love of God to Man - - 272
**The Hospitals of London** - - - 273
**The Doctor's Pulpit.**—In Nature's School - - 275
**Palmistry** - - - 276
**Earth Voices** - - - 276
**The Story of the Insane from Year to Year.**—Asylum Reports - - - 277
**Notes and News** - - - 278
**Everybody's Page** - - - 280
**Annotations.**—Combining Clerks—The Want of Money—The Revolt of the General Practitioner - - - 281
**The Hospital Workshop.**—No. VII. Out-patients at St. Thomas's - - - 282
**Her Heart's Mistake.** By E. D. Cumming. - - - 283
**Metropolitan Hospital Sunday Fund, 1889.**—Awards and Criticisms - - - 285
**In a Hospital** - - - 286
**Amusements and Relaxation** - - - 286
**Scraps and Gleanings** - - - 286

**EXTRA SUPPLEMENT—THE NURSING MIRROR.**

**En Passant.**—Nurse-Stewardesses—The Dangers of a Register—A Warning to Nurses—Harriett Brownlow Byron—Another Parish Nurse—Norwich News—A Sunderland Echo—A Nurse's Soporific—The Red Cross in Holland - lxvii
**Physiology for Nurses.**—Baths and their Effects - lxviii
**Nursing at Nottingham** - - - lxix
**Everybody's Opinion.**—The Pension Fund—Registration - lxix
**Notices—The Nurse's Bookshelf—Presentation** - lxx
**Appointments—Vacancies** - - - lxx
**Notes and Queries** - - - lxx

